# IFN-**γ** signaling stimulates intestinal crypt hyperplasia in celiac disease

**DOI:** 10.1172/JCI198412

**Published:** 2025-10-15

**Authors:** Alexa R. Weingarden

**Affiliations:** Department of Medicine, Division of Gastroenterology, Hepatology, and Nutrition, University of Minnesota, Minneapolis, Minnesota, USA.

## Abstract

Celiac disease, an enteropathy driven by a maladaptive immune response to dietary gluten, is marked by increased proliferation in intestinal crypts, or crypt hyperplasia. However, it is unknown whether this phenomenon is a compensatory response to loss of villus epithelial cells or if it is driven by independent mechanisms. In this issue of the *JCI*, Stamnaes et al. demonstrated that in untreated celiac disease, crypt cells had increased expression of proteins involved in the IFN response, with decreased expression of fatty acid metabolism pathways. These expression patterns were recapitulated in mice treated with IFN-γ, but not mice with intestinal epithelial cell–specific knockout of the IFN-γ receptor. The findings suggest that crypt cells were reprogrammed directly by IFN-γ signaling, independent of changes to epithelial villi.

## Crypt hyperplasia in celiac disease

Celiac disease is a common condition characterized by an abnormal CD4^+^ T cell response to gliadin and related proteins from wheat, barley, and rye (1). Affected individuals carry a specific set of HLA haplotypes — HLA-DQ2 and -DQ8 — which are required for pathological presentation of deamidated gliadin peptides to T cells (2). Upon recognition of their cognate antigen, gliadin-specific CD4^+^ T cells release IFN-γ. This ultimately results in tissue destruction in the intestinal epithelium, driving symptoms and malnutrition in patients (2).

**Figure 1:**
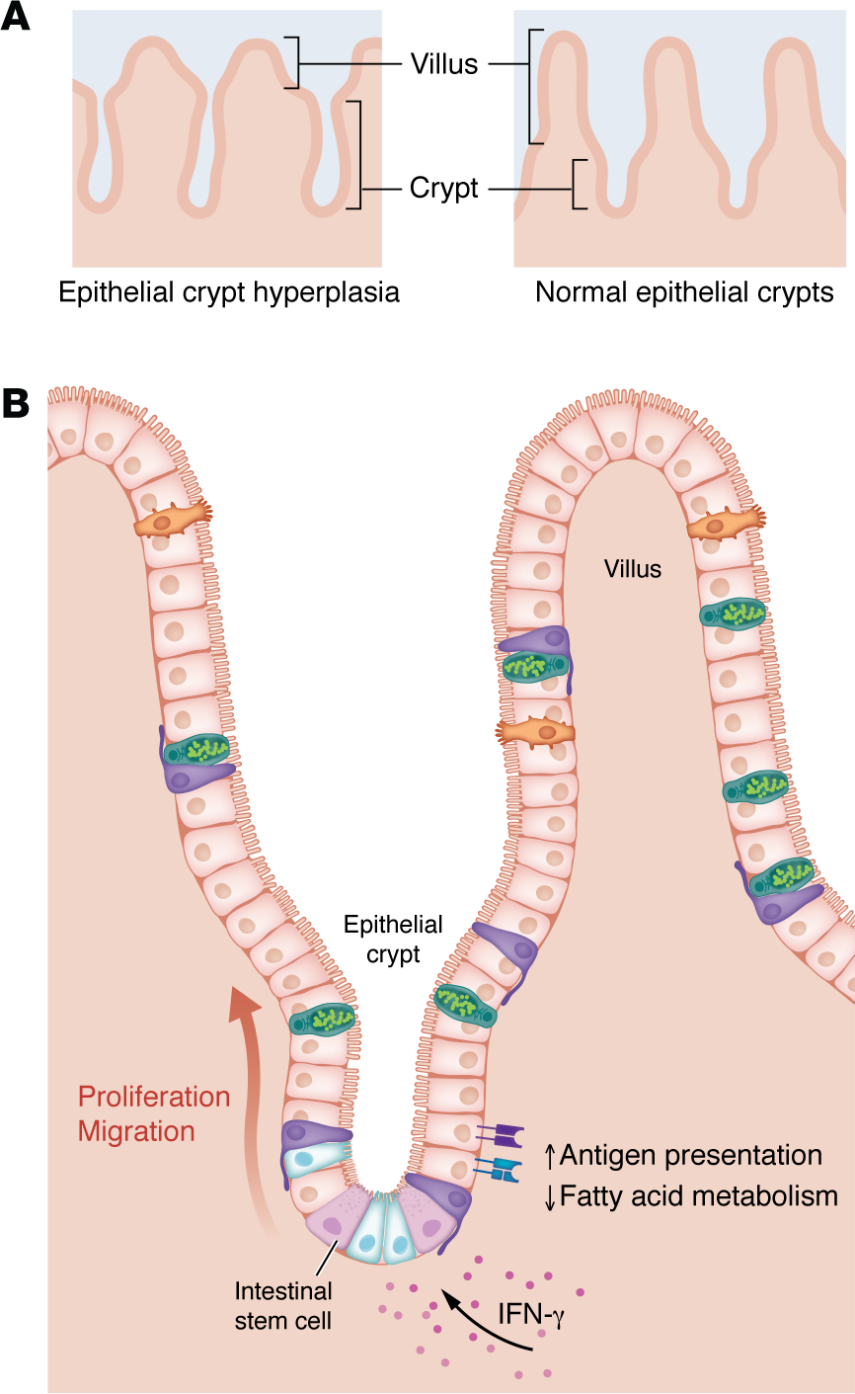
Epithelial crypt hyperplasia is driven by IFN-γ. (**A**) In celiac disease, intestinal villi shorten, and crypts deepen relative to healthy intestinal epithelium. (**B**) Stamnaes et al. showed that in both humans with celiac disease and mice treated with IFN-γ, epithelial crypts had increased cellularity and expression of proteins involved in the IFN-γ response and antigen presentation, with a concomitant decrease in fatty acid metabolic pathways. IFN-γ treatment also induced an increase in migration of epithelial cells toward villi. These changes were reversed with IEC-specific knockout of the IFN-γ receptor 1 (6).

The histological hallmark of celiac disease is loss of absorptive area of the small intestine, denoted by decreased height of intestinal villi and an accompanying increase in the depth of and cellular proliferation in the crypt region (Figure 1A) (3). Prior work has demonstrated that these histological changes can be driven by IFN-γ in both mouse models and human celiac organoids (4, 5). However, it is unclear how these changes are interrelated: Does epithelial destruction in villi induce a responsive hyperproliferation in crypts, or are crypt cells, including intestinal stem cells (ISCs), independently responding to IFN-γ signaling?

## Response to IFN-γ in celiac crypts

In this issue, Stamnaes et al. (6) investigated this question using proteomics analysis of microdissected epithelial crypts from patients with untreated celiac disease (UCeD), patients after treatment with a gluten-free diet (TCeD), and nonceliac control patients. This technique allows for the isolation and analysis of epithelial stem cells and surrounding Paneth and daughter cells and minimizes contributions from villus epithelium or underlying lamina propria. In UCeD, differentially expressed proteins in epithelial crypts were predominantly those related to the IFN-γ response and antigen presentation (Figure 1B). These included CD74, TAP1, and TAP2, HLA molecules, and STAT1, which coordinates transcriptional responses to IFN-γ. Intriguingly, HLA-DQ was not commonly expressed in intestinal epithelial cells (IECs), even in UCeD. Epithelial crypts in UCeD also demonstrated an altered metabolic profile, with downregulation of proteins involved in fatty acid metabolism, PPAR signaling, and retinoid metabolism. Notably, several of these proteins were negatively correlated with expression of the IFN-γ response and antigen presentation proteins, including STAT1 and CPT1A, HLA-DRA and HMGCS2, and TAP1 and FABP1.

TCeD epithelial crypts had proteomic expression patterns more similar to those for the control patients than for patients with UCeD. For example, expression of HMGCS2, STAT1, CD74, TAP1, and HLA-DRA normalized with a gluten-free diet. However, several proteins involved in lipid and retinoid metabolism pathways, including FABP1, APOC3, and RBP2, retained an intermediate expression pattern. In addition, components of immunoglobulin A (IGHA1 and IGJ) were increased in UCeD and remained so to a lesser extent in TCeD, either due to increased transport of IgA through epithelial cells via poly Ig receptor (PIGR) or increased production of IgA from contaminating plasma cells. The authors suggested that this intermediate expression pattern could represent ongoing low-level disease activity even with appropriate treatment of celiac disease.

## IFN-γ signaling in IECs drives crypt hyperplasia

Because IFN-γ response pathways were distinctly upregulated in UCeD, Stamnaes and colleagues sought to examine the effects of IFN-γ on crypt hyperplasia and IECs in mice. Prior work has demonstrated that intraperitoneal injection of IFN-γ drives epithelial villous blunting and crypt hyperplasia (7, 8), emphasizing the importance of this cytokine in celiac disease pathogenesis. Stamnaes et al. (6) found that IFN-γ injection not only led to these histological changes, but also drove changes in the crypt proteome similar to those seen in patients with UCeD (Figure 1). In particular, IFN-γ drove differential expression of CD74, TAP1, RBP2, and other proteins involved in antigen presentation and fatty acid oxidation to a degree that was highly correlated to differential expression of these proteins in patients with UCeD versus control patients.

To further investigate the role of IFN-γ signaling in epithelial crypts, the authors generated a conditional knockout of IFN-γ receptor 1 (*Ifngr1*) in IECs by crossing mice expressing a Cre recombinase under control of the villin promoter (*Villin^Cre^*) to mice with LoxP-flanked *Ifngr1* (*Ifgnr1^fl/fl^*). The resulting animals lacked IFN-γ receptor 1 expression only in IECs (*Ifngr1^IEC–/–^*). Compared with wild-type animals, *Ifngr1^IEC–/–^* mice did not develop crypt hyperplasia and had decreased loss of villous height following IFN-γ injection. Furthermore, expression patterns in antigen presentation and fatty acid metabolism in crypts of wild-type mice treated with IFN-γ were not seen in *Ifngr1^IEC–/–^* mice. Critically, this suggests that crypt hyperplasia in untreated celiac disease is the result of direct IFN-γ signaling in IECs.

## Conclusions and future studies

Through the use of microdissection of epithelial crypts from patients and development of a mouse model with IEC-specific loss of IFN-γ signaling, Stamnaes and colleagues (6) clearly demonstrated the importance of IFN-γ for crypt hyperplasia seen in celiac disease and other small intestine diseases.

One lingering question is the source of IFN-γ. In celiac disease, IFN-γ can be produced by both gliadin-specific CD4^+^ T cells and CD8^+^ cytotoxic T lymphocytes (CTLs), which typically reside in the intraepithelial compartment (9). The authors proposed that because of the predominance of CD4^+^ T cells in the lamina propria, near epithelial crypts, these are more likely to be the source of IFN-γ, rather than CTLs, which cluster near the villus tip in celiac disease (10). This is worth further investigation, as gliadin-specific CD4^+^ T cells producing IFN-γ are not currently thought to be a direct cause of tissue pathology in celiac disease (9).

Whether or not gliadin-specific T cells are directly responsible for pathology in celiac disease, presentation of gliadin peptides to these cells in the intestine seems to be necessary to drive tissue damage (5). Despite the upregulation of antigen presentation machinery in IECs by IFN-γ stimulation, the authors of this study did not find an increase in HLA-DQ expression. This is corroborated by prior work demonstrating that the MHC class II molecules HLA-DR and DP, but not HLA-DQ, are upregulated in celiac disease epithelium (11, 12), but it is distinguished from recent findings that epithelial cells in organoid cultures can present gliadin peptides (13). Rather, in examination of fresh tissue, the authors found that lamina propria CD11c^+^ cells (which could include DCs, plasma cells, or innate lymphoid cells) (14,15) expressed high levels of HLA-DQ, providing evidence that these are most likely to be the critical antigen-presenting cells (APCs) regulating gliadin peptide presentation in intestinal tissue in celiac disease. An important next step could be to identify whether any of these APCs express RORγt, given recent evidence highlighting the importance of this transcription factor in the presentation of food peptides in mice (16–19), although these cells are rare in humans (20). Another critical question is whether upregulation of the nongliadin peptide antigen presentation machinery by IECs has a role in celiac disease pathogenesis.

Finally, the role of fatty acid metabolism in the IFN-γ response in IECs deserves further investigation. Stamnaes et al. found that changes in fatty acid metabolic pathways were inversely correlated to the increase in antigen-presenting and IFN-γ response pathways. In prior work, genetic ablation in ISCs of one enzyme involved in fatty acid oxidation, HMGCS2, which was also decreased in IFN-γ–treated mice, led to ISC dysfunction (21). This suggests that IFN-γ response pathways and fatty acid metabolism are linked in celiac disease and may contribute to epithelial damage, but how these pathways are coregulated remains to be understood.

Overall, Stamnaes et al. (6) have shed light on the regulation of crypt hyperplasia in celiac disease. IFN-γ acts directly on crypt epithelial cells, including ISCs, to drive translational changes in antigen presentation and fatty acid metabolism and ultimately drive epithelial cell proliferation and upward migration (Figure 1). In addition to broadening our understanding of celiac disease pathology, these findings might also have implications for other diseases characterized by crypt hyperplasia.

## Funding support

Life Sciences Research Foundation fellowship and Open Philanthropy grant to ARW.

## Version 1 10/15/2025

Electronic publication
